# Patterns and Drivers of Tree Mortality in Iberian Forests: Climatic Effects Are Modified by Competition

**DOI:** 10.1371/journal.pone.0056843

**Published:** 2013-02-25

**Authors:** Paloma Ruiz-Benito, Emily R. Lines, Lorena Gómez-Aparicio, Miguel A. Zavala, David A. Coomes

**Affiliations:** 1 Department of Forest Ecology and Genetics, Forest Research Center - Instituto Nacional de Investigación y Tecnología Agraria y Alimentaria (CIFOR-INIA), Madrid, Spain; 2 Forest Ecology and Restoration Group, Department of Life Sciences, University of Alcala, Alcalá de Henares, Madrid, Spain; 3 Department of Plant Sciences, University of Cambridge, Cambridge, United Kingdom; 4 Department of Geography, University College London, London, United Kingdom; 5 Instituto de Recursos Naturales y Agrobiología de Sevilla (IRNAS), CSIC, Sevilla, Spain; University of Zurich, Switzerland

## Abstract

Tree mortality is a key process underlying forest dynamics and community assembly. Understanding how tree mortality is driven by simultaneous drivers is needed to evaluate potential effects of climate change on forest composition. Using repeat-measure information from *c.* 400,000 trees from the Spanish Forest Inventory, we quantified the relative importance of tree size, competition, climate and edaphic conditions on tree mortality of 11 species, and explored the combined effect of climate and competition. Tree mortality was affected by all of these multiple drivers, especially tree size and asymmetric competition, and strong interactions between climate and competition were found. All species showed L-shaped mortality patterns (i.e. showed decreasing mortality with tree size), but pines were more sensitive to asymmetric competition than broadleaved species. Among climatic variables, the negative effect of temperature on tree mortality was much larger than the effect of precipitation. Moreover, the effect of climate (mean annual temperature and annual precipitation) on tree mortality was aggravated at high competition levels for all species, but especially for broadleaved species. The significant interaction between climate and competition on tree mortality indicated that global change in Mediterranean regions, causing hotter and drier conditions and denser stands, could lead to profound effects on forest structure and composition. Therefore, to evaluate the potential effects of climatic change on tree mortality, forest structure must be considered, since two systems of similar composition but different structure could radically differ in their response to climatic conditions.

## Introduction

Tree mortality driven by an inability to tolerate harsh environmental conditions is critical for structuring forest communities [1], [2] acting alongside disturbance events such as fires, logging or extreme climatic events to drive regional patterns of species abundance and forest size structure [3], [4]. The ability of a tree to withstand harsh conditions may be strongly modulated by competitive interactions, because when light, water and soil nutrients are preempted by neighbouring trees, it limits the amounts available for effective metabolism [5]–[7]. As a result of climate change, environmental stress and competitive processes will inevitably change across forested landscapes, potentially driving major alterations in tree mortality patterns [8], [9].

Spatial variation in tree mortality across forests landscapes is influenced by multiple interacting factors including tree size, competition and climate (e.g. [10]–[12]). U-shaped size-dependent mortality (i.e. relative high mortality rates in small and large size classes due to light competition and senescence, respectively) has been widely observed in temperate and tropical forests [11], [13]. L-shaped mortality patterns (i.e., high mortality rates of young trees because of competition of limited resources) have been reported in Mediterranean regions, and are typical of stands where dynamics are dominated by competition (e.g. [10], [14]). Competition is a key factor affecting tree mortality, particularly for small trees where competition for light is strong [15]. Moreover, competitive responses could be strongly dependent on species identity of neighbours, and therefore intra- versus inter-specific competition could have different effects on tree performance, growth and mortality [10], [16]. Climatic conditions are known to be fundamental drivers in shaping species distribution and plant performance [17]and increasing frequency and/or intensity of climatic extremes could cause physiological stress and may lead to large-scale increased mortality events (e.g. [8], [18]). A better understanding of the interaction between climatic-driven factors and competition on tree mortality patterns is critically needed to understand likely future successional trajectories [19], particularly in Mediterranean water-limited forests where climatic change effects could be particularly severe [20], [21].

Regional climate change projections consistently predict that the Mediterranean region of Europe will become hotter and drier over the next century [22]. Given that aridity is already a main driver of vegetation patterns across the Mediterranean region [23], the changing climate is predicted to lead to major changes in forest structure and composition [20], [21], [24]. On the other hand, reforestation policies, agricultural land abandonment, coppice cessation and strict protection of some forests have led to a marked increase in forest extent, stand densities and mean tree size over the last 50 years [25]–[27]. High forest density is expected to lead to high mortality rates, due to the inability of many species to tolerate deep shading [28] or water shortage driven by competition [29], as has already been shown for Iberian forests [30]. Even more importantly, changes in climate and competition could interact to exacerbate their individual effects on mortality rates [26], [29]. This possibility has already been suggested for individual tree species such as *Pinus sylvestris* in Mediterranean forests, for which the negative effects of competition for resources (mainly water) were shown to be particulary strong at dry sites [29]. However, no large scale study have yet been undertaken in this region that explores the simultaneous effect of climate and stand structure on tree mortality patterns for multiple coexisting species.

This study quantifies the drivers of mortality for 11 common species in continental Spain using data from 414,841 trees distributed in 35,678 stands, building upon the approach used by Lines *et al.* [11] to quantify mortality trends in US forests. We aimed to (i) quantify the importance of tree species, size, competition and abiotic environmental conditions on tree mortality (excluding fire related and other catastrophic events); and (ii) evaluate how the effect of climate on tree mortality varies along competition gradients. The effects of competition and climate on mortality have been previously addressed in several studies including use of inventory datasets to that end [9], [29], [30]. To our knowledge, however, this study is the first regional multi-species large-scale quantification of tree mortality patterns which considers the interaction between climate and competition.

## Materials and Methods

### Forest Inventory Data

We used permanent plot data from the second and third Spanish Forest Inventory (SFI-2 and SFI-3, recorded in 1986–1996 and 1997–2007 respectively). The inventory consists of permanent plots which sample all forested land in mainland Spain on a 1-km^2^ cell grid [31]. The time interval between SFI-2 and SFI-3 surveys ranged from 6 to 13 years (mean 11.1±0.9 years). SFI plots have a variable-radius design, comprised of four concentric circular subplots of radius 5, 10, 15 and 25 m within which trees within different size classes are monitored. Trees of 7.5–12.4 cm diameter at breast height (*D*) were measured in the 5 m subplot, those of 12.5–22.4 cm diameter in the 10-m radius subplot, those of 22.5–42.4 cm diameter in the 15-m radius subplot, and those larger than 42.4 cm in the 25-m subplot. For each sampled tree, height, *D* and species identity were recorded. From the forest inventory data we derived two variables for each tree which could be considered as drivers of tree mortality: species dominance index and basal area of larger trees. Species dominance index (sdi, proportion) was calculated as the basal area of a species divided by the total stand basal area as an indicator of conspecific abundance and interspecific competition. Secondly, basal area of larger trees (*B_L_*, m^2^/ha) was calculated as the sum of the basal area of trees with greater D within the plot, which is an indicator of asymmetric competition for light [32], [33].

We began our analyses with a dataset of 46,678 plots which included 621,470 trees of 109 species. We then selected 11 species which had at least 10,000 repeat-measure samples (i.e. were recorded as alive in SFI-2 and either alive or dead in SFI-3) and removed all plots in which management such as thinning or harvesting had been recorded during the period between inventories. This subsampling procedure left 414,841 trees of 11 species from 35,678 plots, of which 17% died between inventories. The selected species included three Mediterranean pines (*Pinus halepensis* Miller, *P. pinaster* Ait. and *P. pinea* L.), three mountain pines (*P. nigra* Arnold, *P. sylvestris* L. and *P. uncinata* Mill.), two Mediterranean evergreen oak species (*Quercus suber* L. and *Q. ilex* L.) and three deciduous broadleaved species (*Q. faginea* Lam., *Q. pyrenaica* Willd and *Fagus sylvatica* L.) (see Fig. S1).

### Model Construction and Parameter Estimation

Logistic models of the following general form were used to calculate the annual probability of mortality (e.g. [34], [35]):
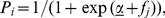
(1)


Where *P_i_* is the annual probability of mortality for tree *i*, 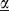 is an intercept term and *f_j_* is a function of predictor variables.

For environmental predictors *X* we considered linear and quadratic functional forms:
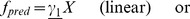
(2)

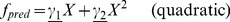
(3)where *X* is a predictor of tree mortality for each species (standardized with mean 0 and standard deviation 1) and 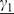 and 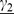 are parameters estimated: 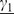 determining the slope of the curve and 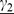 the curvature.

For modeling size-dependence we used an exponential functional form that allows either U-shaped or L-shaped curves depending on the parameter values [11], [19], [36]:
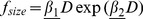
(4)where 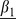 and 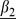 are parameters and *D* is the diameter at breast height. The log-likelihood of the inventory data, given a particular parameter set (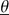) and the model *M* is:



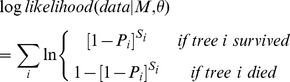
(5)
Eqn (5) represents a sum over all data of the logarithm of the probability of the observation for each tree *i* (survived or died), given *M* and 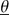, where *S_i_* is the survey interval length (years) for each tree *i*.

Parameters and credible intervals of logistic models were estimated using an adaptive Metropolis-Hastings MCMC algorithm [37] implemented using software written specifically for this study using C for Windows. MCMC is a simple and efficient approach for estimating parameter values and credible intervals which uses likelihood function, and because we did not use informative priors the mean estimated values of the parameters will be similar to those found using maximum likelihood estimation. We used uniform priors for all parameters, with bounds set much wider than expected parameter values (from −6 to 2), and parameter values were initialised at random values close to the middle of the allowable range. We assessed convergence of the parameter estimation by running four parallel chains with different initial parameter values and we used the Gelman-Rubin convergence diagnostic [37] within the *coda* library of R [38]. The models were run using a burn-in of 750,000 iterations for the algorithm to reach the quasi-equilibrium, and a sampling period of 250,000 iterations during which we recorded parameter sets every 100^th^ iteration. This provided us with a set of 2,500 samples from the posterior distribution of the parameter for each model M that we parameterized.

### Variable Selection, Model Comparison and Model Validation

We selected six predictor variables from an initial set of 31 available, in order to focus the analyses on the key drivers of mortality and to avoid convergence problems within the MCMC algorithm associated with including closely correlated variables. The selection of environmental variables was made by performing a Principal Component Analysis in R [39], and retaining variables highly correlated with the two first axis; mean annual temperature (*mat*, °C) and annual precipitation (*ap*, mm) were selected as representative of the climatic conditions for each tree (see Fig. S2 and Appendix S1 for full details). We tested single-predictor models comparing different biotic and competition variables (see Appendix S1) and selected: diameter at breast height (*D*, mm), species dominance index (*sdi*, proportion)) and basal area of larger trees (*B_L_*, m^2^/ha). We fitted models using different edaphic variables as single explanatory variables and retained the single best predictor, a categorical measure of organic matter content (*omc*; taking values of low, medium and high) (see Appendix S1). The final variable set included two climatic variables (*mat*, *ap*), three biotic variables (*D*, *sdi* and *B_L_*), and one edaphic variable (*omc*). All the predictor variables were normalized (i.e. the mean was subtracted from each value and divided by the standard deviation), enabling simple comparisons of effect sizes.

Comparison techniques using Bayesian Information Criterion (BIC [40]), based on the principle of parsimony, were used to decide whether a quadratic function of each variable was needed, or whether a linear function would be sufficient, and whether species-specific parameters could be replaced by cross-species parameters (see Eqns. (3)–(4),). We also explored whether including competition × environment interactions was statistically justified. To determine this, we judged that a model with a BIC that was ten units less than another was considered to have better statistical support. We compared models with different combinations of variables before selecting the best-supported model parameterised using data from all 109 species (Table S2).

### Evaluating the Relationship between Explanatory Variables and Tree Mortality

Changes in mortality rates along environmental gradients were predicted from the best-supported model for each of the 11 most abundant species (following the method used in [11]). To examine the influence of each predictor we created datasets within which all but the predictor of interest was held constant (at each species’ mean value, see Table S3), and we calculated predicted mortality using 100 randomly chosen posterior parameter sets. We plotted these predictions by grouping them into equal size bins which spanned the environmental gradient of interest, and plotting the mean and 95% credible interval of mortality rates of stems in the bin.

To examine the interaction between competition and climate, and evaluate how the effect of climate on tree mortality varied along competition gradients, we predicted the response of trees to competition (B_L_) in ‘cool’ and ‘hot’ locations (i.e. using the lower and upper quartiles of mean annual temperature for a species when making the prediction; see Table S4) and ‘dry’ and ‘wet’ locations (i.e. using lower and upper quartiles of mean rainfall for a species when making the prediction; see Table S4). As before, the rest of the predictor variables were maintained at their mean value for each species.

## Results

### Effects of Tree Size, Competition and Abiotic Variables on Tree Mortality

The best-supported model included the effects of all predictor variables as species-specific (see Table S1–S2 for comparisons of candidate models) and took the form:
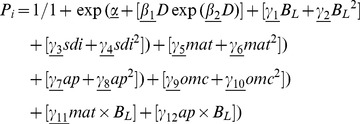
(6)where predictor variables were: diameter at breast height (D), basal area of larger trees (B_L_), species dominance index (sdi), mean annual temperature (mat), annual precipitation (ap), and organic matter content (omc) (see Table S5 for fitted parameter values). Note that D is modelled in exponential form and the rest of predictor variables were all modelled as quadratic functions rather than linear ones. The best model also included interactions between competition and annual precipitation, and between competition and mean annual temperature.

BIC model comparisons indicate that tree size (D) and asymmetric competition (B_L_) were the best determinants of tree mortality for seven of the eleven species analysed (Table 1). Climatic variables were generally less important, with mean annual temperature having a larger influence than annual precipitation, whilst species dominance index and soil organic matter content explained relatively little of the deviance (Table 1).

**Table 1:** Relative importance for six variables as predictors of tree mortality, based on Bayesian Information Criterion (BIC) values of single-variable models.

Species name	Abbreviation	N	Variable Rank	Predictor variables (ΔBIC)					
				D	B_L_	sdi	ap	mat	omc
*P. halepensis*	*Pha*	49,297	B_L_>>D>>omc>sdi>>mat>>ap	212	0	277	311	289	268
*P. pinea*	*Ppe*	15,538	D>B_L_>>sdi>ap> mat>omc	0	7	130	135	139	140
*P. pinaster*	*Ppa*	75,785	B_L_>>D>>ap>> omc>>sdi>>mat	30	0	805	761	937	794
*P. nigra*	*Pni*	45,847	mat>>D>>omc>>B_L_>>sdi>>ap	1,315	1,580	1,813	1,840	0	1,529
*P. sylvestris*	*Psy*	87,320	B_L_>>D>>mat>>omc>>sdi>>ap	1,337	0	1,882	1,927	1,727	1,839
*P. uncinata*	*Pun*	10,750	B_L_>>ap>> mat>sdi>D>omc	76	0	76	55	69	82
*Q. ilex*	*Qil*	56,850	D>>B_L_>>sdi>>mat>>ap>> omc	0	135	162	226	215	248
*Q. suber*	*Qsu*	16,593	D>>mat>B_L_>>sdi>ap> omc	0	23	37	38	19	42
*Q. pyrenaica*	*Qpy*	23,789	D>>B_L_>>mat>omc>sdi>>ap	0	121	407	421	398	404
*Q. faginea*	*Qfa*	12,008	sdi>D>B_L_>>mat>>omc>>ap	7	9	0	64	20	45
*F. sylvatica*	*Fsy*	21,064	B_L_>D>>omc>sdi>mat>ap	3	0	62	69	69	57

ΔBIC values are shown for each predictor variable for each species, which is the difference between the best-supported model and the model of interest. The “variable ranking” column ranks the models in order of statistical support (i.e. BIC value); models considered much less strongly supported than a higher ranking model (i.e. with ΔBIC >10 compared with the model ranked immediately above it) are indicated with “>>” while similarly supported models are indicated by “>”. Sample size (N) and ΔBIC for each species and each predictor is shown: diameter at breast height (*D*), basal area of larger trees (B_L_), species dominance index (sdi), mean annual temperature (mat), annual precipitation (ap) and organic matter content (omc).

**Figure 1:**
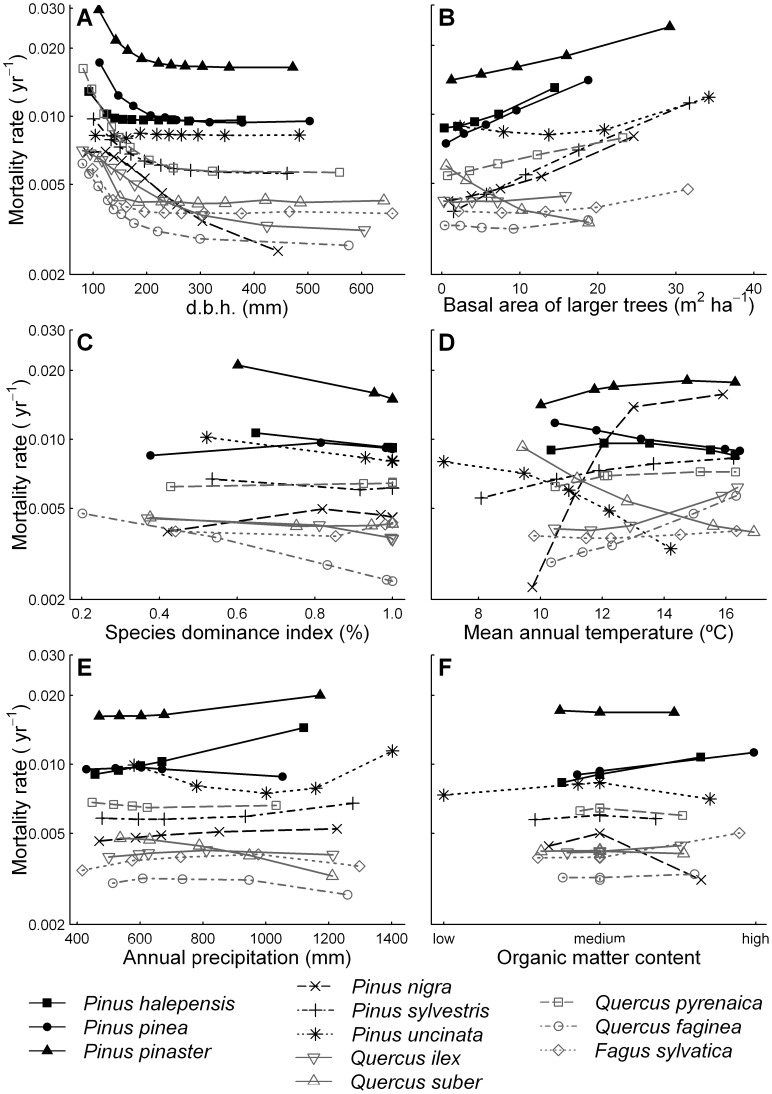
Predicted mortality against stem size, competition and environmental gradients. Predicted mortality rates (yr^−1^, log scale) in relation to (**A**) diameter at breast height (mm), (**B**) basal area of larger trees (m^2^ ha^−1^), (**C**) species dominance index (percentage), (**D**) mean annual temperature (°C) and (**E**) annual precipitation (mm) for the 11 most common species of the Iberian Peninsula.

Pine mortality was most strongly influenced by B_L_, whilst oak species’ mortality was highly size dependent (Table 1). For most species, mortality rates were greatest for small trees (D <200 mm), decreased rapidly with size, before leveling out at larger tree sizes (Fig. 1a). The two exceptions to this rule were *P. uncinata*, for which the mortality rate was virtually invariant of tree size, and *P. nigra*, for which mortality continued to decrease with size without leveling off (Fig. 1a). Tree mortality generally increased with the basal area of larger trees, with this increase being particularly strong for pine species and *Q. pyrenaica* (Fig. 1b and Table S5). For most species the mortality response showed a stronger response at high B_L_ (Fig. 1b). Mortality rates of most species (seven out of 11; see negative value of parameter 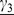, Table S5) decreased as their dominance in a stand increased, and this effect was particularly strong for Mediterranean pines and the broadleaved species *Q. faginea* and *Q. ilex* (Fig. 1c).

Eight of the 11 species studied had higher annual mortality rates with increasing mean annual temperature (all but *P. pinea*, P. *uncinata* and *Q. suber*; Fig. 1d), and the effect was particularly strong for *P. nigra* (see also Table 1). Most species experienced higher increases in mortality at the lower part of the temperature gradient than at high temperatures (but see *Q. ilex* and *Q. faginea*; Fig. 1d). For most species, annual precipitation had a lower effect on tree mortality than mean annual temperature (Fig. 1d,e), and seven of the 11 species showed increasing tree mortality along the precipitation gradient (see positive parameters 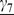; Table S5), especially pines (Fig. 1e). For the four remaining species (*Q. suber*, *P. pinea*, *Q. faginea* and *Q. pyrenaica*), mortality decreased as precipitation increased. The effect of soil organic matter content on tree mortality was relatively low for most species, and much less important than the effect of climate (Fig. 1f, Table 1).

### Climate Effects on Tree Mortality Along Competition Gradients

**Figure 2:**
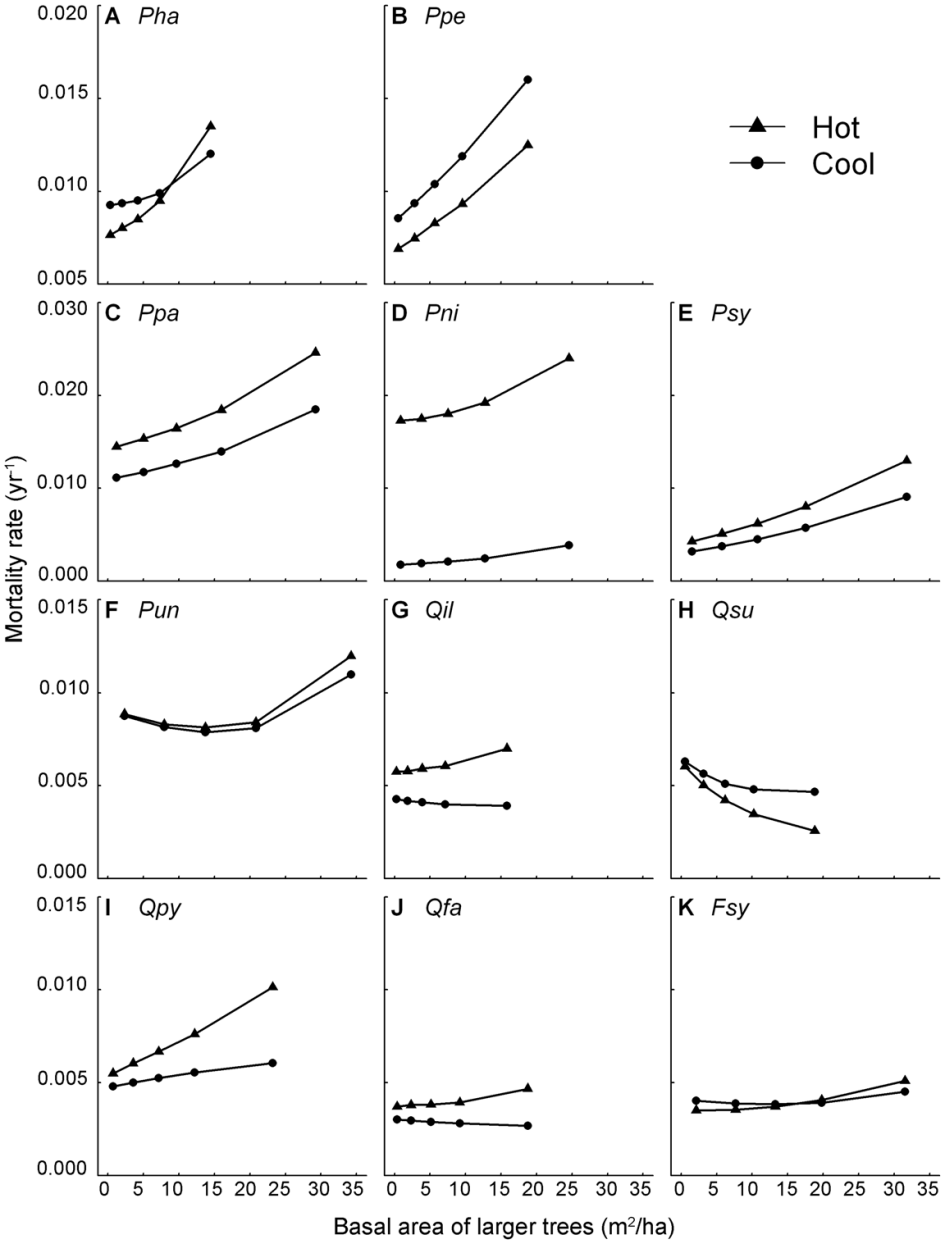
Predicted mortality in relation to B_L_ in two contrasting climatic areas based on temperature. Predicted annual mortality rates (yr^−1^) in two contrasting mean annual temperature areas (hot vs. cool) along basal area of larger trees (m^2^ ha^−1^) for the 11 most common species of the Iberian Peninsula: (**A**) *P. hapelensis* (*Pha*), (**B**) *P. pinea* (*Ppe*), (**C**) *P. pinaster* (*Ppa*), (**D**) *P. nigra* (*Pni*), (**E**) *P. sylvestris* (*Psy*), (**F**) *P. uncinata* (*Pun*), (**G**) *Q. ilex* (*Qil*), (**H**) *Q. suber* (*Qsu*), (**I**) *Q. pyrenaica* (*Qpy*), (**J**) *Q. faginea* (*Qfa*), (**K**) *F. sylvatica* (*Fsy*).

**Figure 3:**
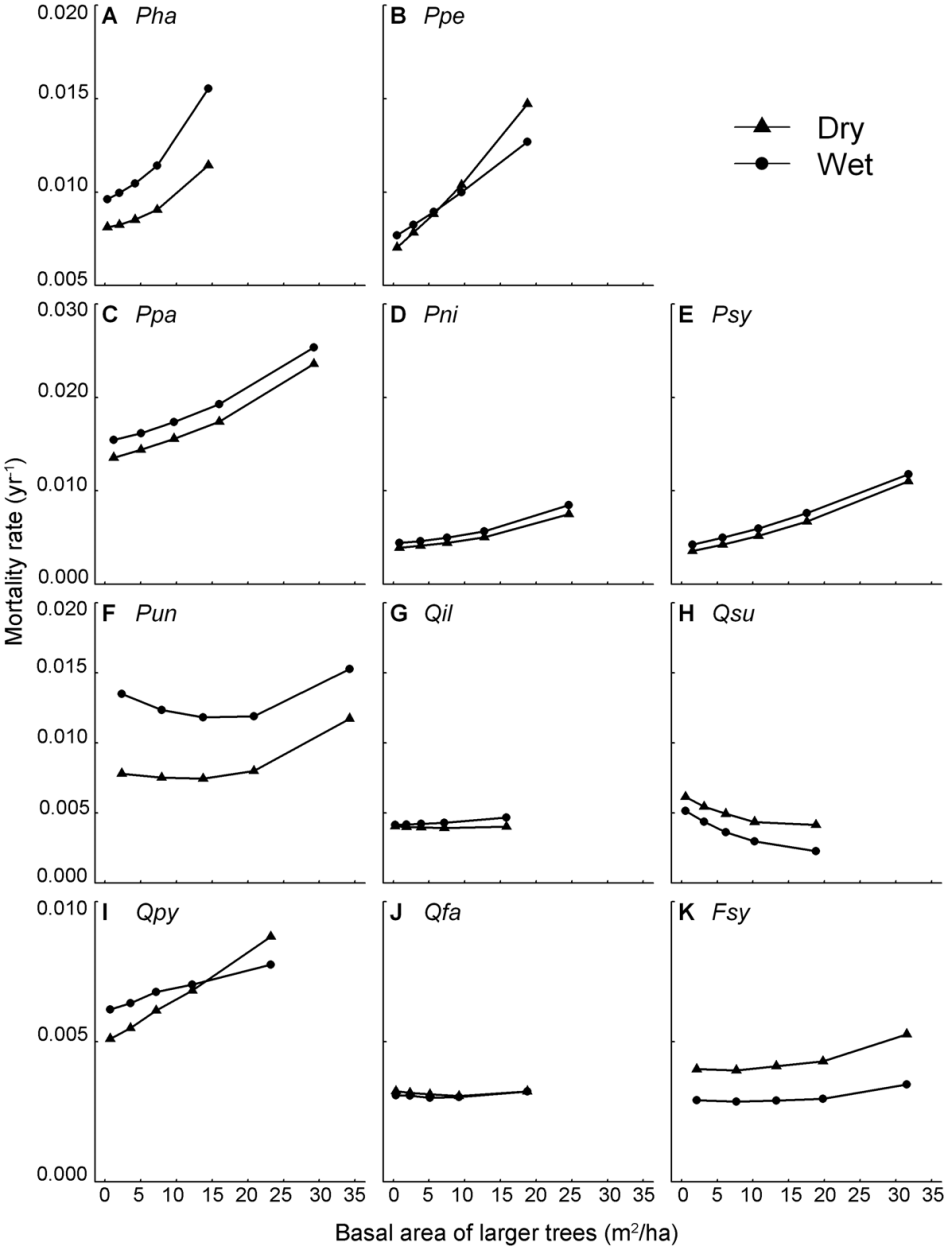
Predicted mortality along B_L_ in two contrasting climatic areas based on precipitation. Predicted annual mortality rates (yr^−1^) in two contrasting annual precipitation areas (wet vs. dry) along basal area of larger trees (m^2^ ha^−1^) for the 11 most common species of the Iberian Peninsula: (**A**) *P. hapelensis* (*Pha*), (**B**) *P. pinea* (*Ppe*), (**C**) *P. pinaster* (*Ppa*), (**D**) *P. nigra* (*Pni*), (**E**) *P. sylvestris* (*Psy*), (**F**) *P. uncinata* (*Pun*), (**G**) *Q. ilex* (*Qil*), (**H**) *Q. suber* (*Qsu*), (**I**) *Q. pyrenaica* (*Qpy*), (**J**) *Q. faginea* (*Qfa*), (**K**) *F. sylvatica* (*Fsy*).

The modulation of environmental conditions by competitive interactions is described in the temperature × competition and rainfall × competition terms in Eqn (6) and values of the γ_11_ and 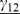 parameters in Table S5. To illustrate the influences of these interactions on mortality we plotted predicted mortality rates against B_L_ for trees growing in cool vs. hot sites (Fig. 2) and wet vs. dry sites (Fig. 3). Differences in tree mortality between cool and hot sites were more pronounced at high B_L_ suggesting that competition magnifies the effects of climate on tree mortality (Fig. 2). We generally found a similar pattern for annual precipitation, suggesting that competition inflates the effects of precipitation on tree mortality, increasing the effect of asymmetric competition in wetter sites in five of the six pine species studied (except for *P. uncinata* and *Q. faginea*, Fig. 3).

## Discussion

### Effects of Tree Size and Competition on Tree Mortality

Our analysis showed that tree mortality in Iberian forests was largely driven by competition and tree size, confirming that these variables have a relatively stronger impact on mortality than abiotic variables [10], [29], [41]. Tree mortality decreased and then leveled off with size (L-shaped mortality), with no species showing increased mortality rates at sizes larger than 200 mm (U-shaped mortality curves, which are commonly reported [11], [19], [42]). L-shaped mortality patterns suggest that most stands are relatively young and still at the stem exclusion phase of development (e.g. [43], [44]), and that few stands had reached the senescence phase [45]. Our results are typical of young competitively thinning stands within which small suppressed trees have high mortality rates [14], [30]. This may be the case for many Iberian forests that are either at an early successional stage (i.e. after land abandonment during the last decades) or in which silvicultural exploitation ceased (i.e. cessation of charcoal coppicing) [46], [47].

Mortality rates of most species increased because of asymmetric competition (Fig. 1b), suggesting strong effects of asymmetric competition for light on small suppressed trees [43], [45]. Interestingly, the increases of mortality along competition gradients were not linear, and most species experienced the largest increases in mortality at high competition levels. Intense competition for limiting resources could push species to their physiological limit and result in the non-linear responses observed along the basal-area gradients [5], [48]. Pine species were the most sensitive to competition, whereas a less pronounced response was observed for broadleaved species (particularly Mediterranean evergreen oaks, Fig. 1b). These results agree with previous studies showing Mediterranean pine species to be highly sensitive to competition from neighbours, probably because they are relative shade-intolerance [16], [49], [50]. Oak species have a comparatively higher ability to survive and grow in low light than pine species [28], and so are less dependent on competition for light [16]. The slight or even negative response observed for Mediterranean evergreen oaks could be related with the facilitative or protective effect of neighbours, which has already been observed for relatively large trees [19].

Tree mortality was influenced by conspecific abundance (as indicated by species dominance index), and this effect was strong for Mediterranean pine species and the broad-leaved *Q. faginea* and *Q. ilex* (Table 1 and Fig. 1c). Generally, we observed a decrease in mortality with increasing species dominance index (i.e. at high intraspecific competition, Fig. 1c). Other studies have found that the identity of neighbour species can strongly affect the growth and mortality of a target tree species [10], [16], [51]. Characteristics of neighbours are critical for tree mortality rates because of their differential effects on the competitive environment experienced [52], [53]. The fact that pine species generally more affected by conspecific abundance than oaks could be related to their successional status in Mediterranean forests (see negative 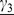 parameter value, Table S5). Thus, whereas pines can be the successional end point under severe environmental conditions [54], [55], in more mesic environments they usually represent early successional stages, and are eventually partially or totally displaced by oak species. Oak species are comparatively more shade tolerant than pines, accumulate more leaf area and form denser crowns which cast deeper shade [7], [56], [57]. Therefore, mortality rates of shade-intolerant pines could be expected to be lower in monospecific stands where they do not have to compete for light with competitively superior oak species.

### Effects of Climate on tree Mortality

We found that tree mortality rates generally increased with increasing temperature (Fig. 1d). Increases in mortality with temperature were not restricted to the hotter parts of the gradient, and we found steep increases in cooler areas for some species (Fig. 1d, [8], [29]). The negative effects on mortality at high temperatures are probably related to the strong influence that temperature-driven stress has in Mediterranean areas [2], [9]. At high temperatures, pines close stomata to prevent hydraulic failure (i.e. cavitation of water columns), and this can lead to carbon starvation because of depletion of carbon stores due to high respiration costs [8], [18]. Meanwhile, oaks have drought tolerance mechanisms (i.e. stomatal closure is less severe and transpiration continues at relatively high rates) and are therefore more likely to die because of hydraulic failure rather than carbon starvation, although both processes are strongly interrelated [58], [59]. In agreement with this idea drought-induced mortality has already been reported for Iberian mountain pine populations of *P. nigra* and *P. sylvestris* at the rear edge of their distribution [60]. The exception to the general pattern was found with the mountain pine *P. uncinata* and the Mediterranean species *P. pinea* and *Q. suber*, for which we observed decreased mortality with increased temperatures (Fig. 1d). *P. uncinata* largely occupies cold areas in the high mountains of the Iberian Peninsula, where low temperatures represent a physiological constraint for species performance. Therefore, this species could benefit from the more benign conditions derived from an increase in mean annual temperature (Fig. S1f, [46]). Such an increase could also benefit particularly cold-sensitive species, such as Mediterranean *P. pinea* and *Q. suber* [61].

Annual precipitation had relatively lesser effect on tree mortality for most species (Table 1, Fig. 1, [9], [41]). Generally, the mortality rates of pine species were more affected by precipitation than broad-leaved species and increased at high precipitation levels (Fig. 1e). The increased mortality in wet sites, although in principle counter-intuitive for water-limited environments like the Mediterranean, could be related to other co-occurring abiotic conditions in these more productive sites. Water availability correlates with favourable conditions for growth and thus could lead to high mortality of small and suppressed trees [30], [62]. Another plausible explanation is that high precipitation levels in Iberian forests usually occur in areas of high altitude and low temperature, where increased mortality rates may be caused by the low tolerance of tree species to cold conditions or frost damage [61].

### Climatic Effects on Tree Mortality Along Competition Gradients

A major finding of this work is the importance of the interactive effects of climate and competition on the mortality rates of the 11 tree species studied. These imply that the differences in mortality rates among contrasting climatic scenarios (particularly those of temperature) are strongly amplified by high competition levels for most species. For example, we found that while mortality rates of *Q. pyrenaica* in hot and cold areas were relatively similar in the absence of competition (a rate of around 0.005 trees per tree per year), mortality in hot areas was almost double that in cold sites at high competition levels (Fig. 2i). Moreover, for some species the climate-competition interaction caused complete changes in mortality rates from being higher at low competition levels in cool than in hot sites, and observing the contrary trend at high competition levels (e.g. see *P. halepensis* and *F. sylvatica* in Fig. 2). The high mortality rates in hot sites at high competition levels could be the result of the combination of increases in tree water demand due to higher evapotranspiration rates and lower water supply due to increased competition with neighbours [26], [48]. On the other hand, precipitation effects on tree mortality were also aggravated at high competition levels for five of the six pine species studied, highlighting that asymmetric competition is particularly strong in wetter and more productive sites (Fig. 3). Thus, in agreement with previous studies performed on tree growth [63], we suggest competition for light could be particularly intense in productive areas, causing a general increase in tree mortality rates of pine species. Our findings imply that predictions of climate change effects on forest dynamics and species distribution cannot be made without acknowledging the particular structural characteristics of the forest, since two systems of similar composition but different structure could radically differ in their response to changes in climate.

Interestingly, the nature of the climate and competition interaction found in this study for tree mortality is opposite to that found in a parallel study for tree growth [16]. While we found that competition amplified mortality differences among contrasting climatic areas, Gómez-Aparicio *et al.* [16] found that competition limited growth rates and their variation along climatic gradients. When put together, the results of these two studies suggest that increased aridity in Mediterranean regions could cause strong changes on tree demographics of high-density forests by both increasing mortality and suppressing growth. Moreover, since the predicted changes are not the same for all species, this could translate into larger inter-specific differences in these key demographic traits, potentially altering competitive hierarchies and affecting forest structure and composition.

### Conclusions

Our results suggest that tree mortality in Iberian forests is primarily shaped by tree size and competition, but is also influenced by climatic conditions (particularly temperature) and soil properties. We show, for the first time, that competition and temperature interact strongly to determine tree mortality rates, with competition causing an amplification of climate effects for almost all species (i.e. negative effects of high mean annual temperature). These interactions imply that dense forests are particularly vulnerable to large increases in mortality rates due to climate change. This result is important for the Mediterranean region as dense forests are becoming increasingly common as a result of reforestation policies and less intensive exploitation of forest resources [47], [54]. Density reductions, either by self-thinning or selective thinning of suppressed trees could be used as an adaptation measure to climatic change in highly dense stands of both pines and hardwoods, since we found that virtually all tree species were strongly affected by competition either directly (in the case of pines) or indirectly through the interaction with climate (in the case of broadleaved species). This is particularly true for forest systems in which current density is a legacy or previous management history rather than a natural secondary succession process (i.e., abandonment of old pine plantations and coppices). Moreover, basal area reductions in these forest types would not only reduce tree mortality and increase tree growth [12], [16], but might also have other desirable effects such as promoting tree and understory species regeneration (e.g. [27]), species coexistence [64] and carbon accumulation (e.g. [30]); and reducing fire risk [65], defoliation [9] and forest decline [66], [67].

The fact that mortality of Mediterranean tree species is shaped by the interaction among competition and climate suggests that changes in demographic and competition processes are likely to be key factors shaping future species distribution and forest structure in the Mediterranean region [9], [16]. A direct implication of this result is that any attempt to predict changes in species distributions due to climate change should not only be based on individual species responses to climate (e.g. [68]), but also consider stand structure characteristics and demographic processes (see also [16]). Our results show that interactions between climate and competition could intensify the effects of global change on the key demographic process of mortality, and highlight the importance of considering these interactions in management practices and predictions of future species distribution.

## Supporting Information

Figure S1 **Map of the stands included for each one of the 11 species included in the analysis.** The permanent stands of the SFI used to analyze the mortality patterns in the Iberian Peninsula with the total number of trees (n) and the death trees (m) for each species are shown. In black the *Pinus* species and in grey the *Quercus* and *Fagus* species are shown.
(TIF)

Figure S2 **Principal Component Analysis if topographic and climatic variables.** Results of principal component analysis performed over the initial 26 topographic and climatic variables showing the graph of **(A)** PC1 in relation to mean annual temperature (°C) and **(B)** PC2 in relation to annual precipitation (mm). The red line shows a linear adjustment between the PCA axis and the climatic variable.
(TIF)

Table S1 **Comparison of single-predictor models fitted for each of the six predictors of tree mortality; models were fitted which allowed parameters to vary among species and compared with models in which a single parameter was fitted for all species; different functional forms are all compared.**
(DOC)

Table S2 **Comparison of alternative mortality models specified at species level.** We tested 16 candidate models without interaction and non-linear variables and seven models that include all possible interactions between climatic variables and basal area of larger trees (testing linear and non-linear forms of the climatic variables).
(DOC)

Table S3 **Mean values for each numeric variable and species included in the final mortality model.** Standard error, minimum (Min) and maximum (Max) ([Min, Max]) is also given.
(DOC)

Table S4 **Temperature and precipitation in cool vs. hot and dry vs. wet sites, respectively.** Mean [minimum, maximum] values of mean annual temperature in cool and hot sites and annual precipitation in dry and wet sites are given for the 11 species included in the best model.
(DOC)

Table S5 **Bayesian means and 95% credible intervals for each parameter of the final model for each of the 11 common species.**
(DOC)

Appendix S1 **Description of biotic and abiotic variables included in the analysis and variable selection.**
(DOCX)
