# Supplementary figures and images for: Patterns and Drivers of Tree Mortality in Iberian Forests: Climatic Effects Are Modified by Competition
Source: PLoS One. 2013 Feb 25;8(2):e56843. doi: 10.1371/journal.pone.0056843 (PMC3581527; doi:10.1371/journal.pone.0056843)

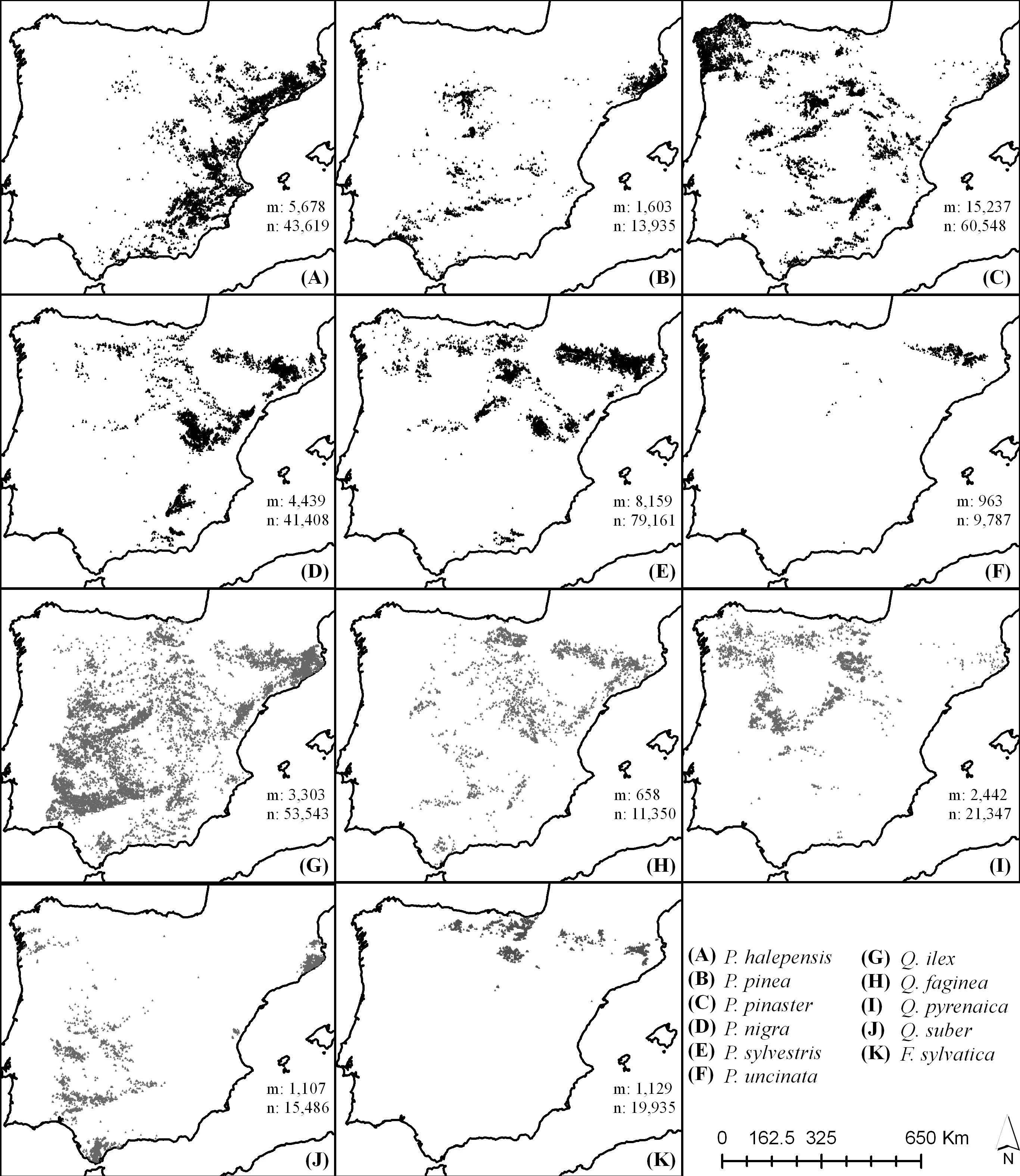

Supplement: Figure S1 — Map of the stands included for each one of the 11 species included in the analysis. The permanent stands of the SFI used to analyze the mortality patterns in the Iberian Peninsula with the total number of trees (n) and the death trees (m) for each species are shown. In black the Pinus species and in grey the Quercus and Fagus species are shown. (TIF) [file pone.0056843.s001.tif]

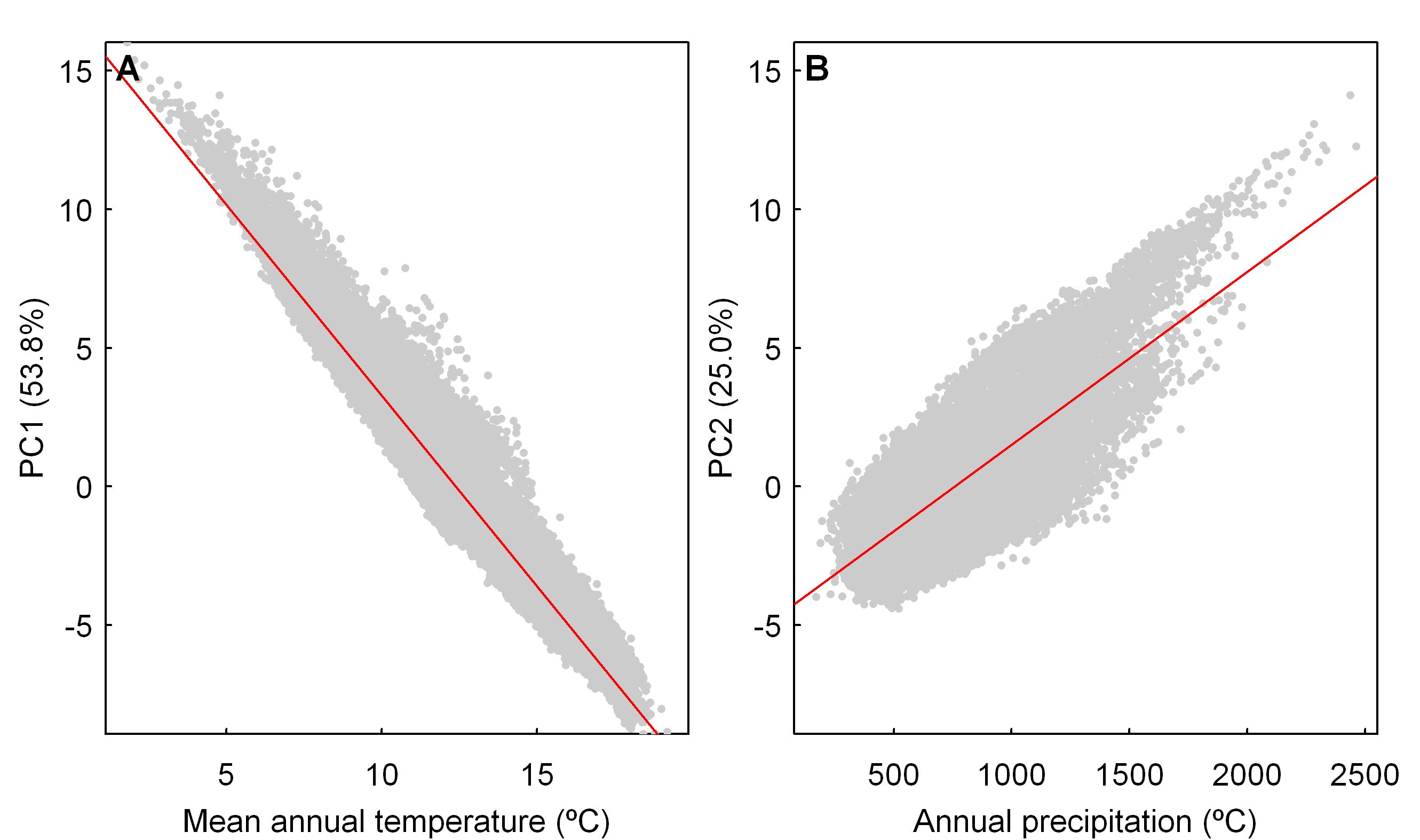

Supplement: Figure S2 — Principal Component Analysis if topographic and climatic variables. Results of principal component analysis performed over the initial 26 topographic and climatic variables showing the graph of (A) PC1 in relation to mean annual temperature (°C) and (B) PC2 in relation to annual precipitation (mm). The red line shows a linear adjustment between the PCA axis and the climatic variable. (TIF) [file pone.0056843.s002.tif]
