# Supplementary material for: Patterns and Drivers of Tree Mortality in Iberian Forests: Climatic Effects Are Modified by Competition
Source: PLoS One. 2013 Feb 25;8(2):e56843. doi: 10.1371/journal.pone.0056843 (PMC3581527; doi:10.1371/journal.pone.0056843)
Supplement: Table S1 — Comparison of single-predictor models fitted for each of the six predictors of tree mortality; models were fitted which allowed parameters to vary among species and compared with models in which a single parameter was fitted for all species; different functional forms are all compared. (DOC) [file pone.0056843.s003.doc]

**Table S1. Comparison of mortality models parameterised with one predictor variable.** Comparison of BIC for the 22 models parameterised with one predictor variable, using each of the six potential predictors in their linear and non-linear form at forest and species level (except for *D* that was included in exponential form).

|  | Linear,  forest level | Exponential (*D*) or non-linear,  forest level | Linear,  species level | Exponential (*D*) or non-linear, species level | ∆ BIC | ∆BIC |
| --- | --- | --- | --- | --- | --- | --- |
| Model | (NP: 2) | (NP: 3) | (NP: 22) | (NP: 33) | (SNL-SL) | (FNL-SNL) |
| D |  | 342004 |  | 272289 |  | 69715 |
| B_L_ | 337428 | 337372 | 271242 | 271185 | 57 | 66187 |
| Sdi | 343511 | 343497 | 275068 | 274962 | 106 | 68535 |
| Mat | 343023 | 342165 | 274922 | 273191 | 1731 | 68973 |
| Ap | 343557 | 343196 | 275234 | 275158 | 75 | 68038 |
| Omc | 343431 | 343413 | 275145 | 274759 | 386 | 68654 |

It is shown the number of parameters (NP) of each model group and the ∆BIC between the linear and non linear model at species level (SNL-SL) and the ∆BIC between the forest level and species level in non-linear form (FNL-SNL) for each predictor variable: diameter at breast height (*D*), basal area of larger trees (B_L_), species dominance index (sdi), mean annual temperature (mat), annual precipitation (ap) and organic matter content (omc).
