# Supplementary material for: Patterns and Drivers of Tree Mortality in Iberian Forests: Climatic Effects Are Modified by Competition
Source: PLoS One. 2013 Feb 25;8(2):e56843. doi: 10.1371/journal.pone.0056843 (PMC3581527; doi:10.1371/journal.pone.0056843)
Supplement: Table S2 — Comparison of alternative mortality models specified at species level. We tested 16 candidate models without interaction and non-linear variables and seven models that include all possible interactions between climatic variables and basal area of larger trees (testing linear and non-linear forms of the climatic variables). (DOC) [file pone.0056843.s004.doc]

**Table S2. Comparison of alternative mortality models specified at species level.** We tested 16 candidate models without interaction and non-linear variables and four models that include all possible interactions between climatic variables and basal area of larger trees.

| **Variables** (*D* (exp) and B_L_ (NL) retained) | | | | | **NP** | **∆BIC** |
| --- | --- | --- | --- | --- | --- | --- |
| **sdi** | **mat** | **ap** | **omc** | **Interaction** |
| **NL** | **NL** | **NL** | **NL** | **mat x B_L_+ ap x B_L_** | **165** | **0.0** |
| NL | NL | NL | NL | mat x ap x B_L_ | 154 | 99.4 |
| NL | NL | NL | NL | mat x B_L_ | 154 | 122.7 |
| NL | NL | NL | NL | ap x B_L_ | 154 | 188.2 |
| NL | NL | NL | NL | - | 143 | 162.6 |
| NL | NL | NL | - | - | 121 | 383.5 |
| NL | NL | - | NL | - | 121 | 427.5 |
| - | NL | NL | NL | - | 121 | 592.7 |
| NL | NL | - | - | - | 99 | 696.6 |
| - | NL | NL | - | - | 99 | 772.3 |
| - | NL | - | - | - | 77 | 1,157.8 |
| - | NL | - | NL | - | 99 | 1,683.4 |
| NL | - | NL | NL | - | 121 | 1,854.9 |
| NL | - | - | NL | - | 99 | 2,155.6 |
| NL | - | NL | - | - | 99 | 2,274.1 |
| - | - | NL | NL | - | 99 | 2,296.3 |
| NL | - | - | - | - | 77 | 2,582.5 |
| - | - | - | NL | - | 77 | 2,625.7 |
| - | - | NL | - | - | 77 | 2,710.8 |
| - | - | - | - | - | 55 | 2,969.5 |

Variables are included AS “*NL*” (non-linear form), “*exp*” (exponential form) or “–” (no included), *NP* is the number of parameters. We used all possible combinations of the six tree mortality predictors selected: tree size (*D*), basal area of larger trees (*B_L_*), species dominance index (*sdi*), mean annual temperature (*mat*), annual precipitation (*ap*) and organic matter content (*omc*). The best fitting model is given in ∆BIC value of zero (bold), comparing the full model with models dropping the effect of interactions, species dominance, climate, and organic matter content.
