# Supplementary material for: Patterns and Drivers of Tree Mortality in Iberian Forests: Climatic Effects Are Modified by Competition
Source: PLoS One. 2013 Feb 25;8(2):e56843. doi: 10.1371/journal.pone.0056843 (PMC3581527; doi:10.1371/journal.pone.0056843)
Supplement: Table S3 — Mean values for each numeric variable and species included in the final mortality model. Standard error, minimum (Min) and maximum (Max) ([Min, Max]) is also given. (DOC) [file pone.0056843.s005.doc]

**Table S3.** Mean values for each numeric variable and species included in the final mortality model. Standard error, minimum (Min) and maximum (Max) ([Min, Max]) is also given.

| **(A)** | ***D*** | **B_L_** (m^2^/ha) | **Species dominance index** (%) | **Mean annual temperature** (ºC) | **Annual precipitation** (mm) |
| --- | --- | --- | --- | --- | --- |
| *P. halepensis* | 200.13 ± 0.374 | 5.66 ± 0.0244 | 0.93 ± 0.0008 | 13.92 ± 0.0057 | 551.5 ± 0.6617 |
|  | [75, 990] | [0, 47.27] | [0.02, 1] | [9.95, 19.21] | [170, 1503] |
| *P. pinea* | 260.97 ± 0.9167 | 7.47 ± 0.0566 | 0.84 ± 0.002 | 14.29 ± 0.0162 | 620.1 ± 1.2605 |
|  | [75, 1305] | [0, 56.57] | [0.01, 1] | [9.12, 18.56] | [312, 1682] |
| *P. pinaster* | 247.47 ± 0.3838 | 12.22 ± 0.0383 | 0.91 ± 0.0007 | 12.09 ± 0.0065 | 824.73 ± 1.2124 |
|  | [75, 1146] | [0, 72.99] | [0.01, 1] | [7.69, 18.46] | [322, 3058] |
| *P. nigra* | 210.68 ± 0.4796 | 9.9 ± 0.0424 | 0.84 ± 0.0011 | 10.93 ± 0.0057 | 775.48 ± 1.002 |
|  | [75, 955] | [0, 66.13] | [0.01, 1] | [6.24, 15.62] | [360, 2334] |
| *P. sylvestris* | 230.37 ± 0.3556 | 13.46 ± 0.0382 | 0.89 ± 0.0007 | 9 ± 0.0051 | 965.03 ± 0.7169 |
|  | [75, 1124] | [0, 79.59] | [0.01, 1] | [3.43, 14.72] | [428, 1977] |
| *P. uncinata* | 244.05 ± 1.0659 | 15.8 ± 0.1133 | 0.89 ± 0.002 | 5.77 ± 0.0122 | 1214.89 ± 1.8534 |
|  | [75, 1283] | [0, 77] | [0.01, 1] | [1.81, 14.2] | [521, 1975] |
| *Q. ilex* | 231.56 ± 0.6934 | 5.79 ± 0.0258 | 0.84 ± 0.0011 | 13.15 ± 0.0097 | 736.21 ± 0.8477 |
|  | [75, 1522] | [0, 69.86] | [0.01, 1] | [6.6, 18.67] | [268, 1988] |
| *Q. suber* | 312.24 ± 1.258 | 7.78 ± 0.0536 | 0.81 ± 0.0019 | 15.41 ± 0.011 | 876.82 ± 1.5389 |
|  | [75, 1465] | [0, 50.41] | [0.01, 1] | [10.07, 19.03] | [426, 1756] |
| *Q. pyrenaica* | 208.65 ± 0.9417 | 9.38 ± 0.0556 | 0.87 ± 0.0016 | 10.61 ± 0.0107 | 932.7 ± 1.533 |
|  | [75, 1724] | [0, 66.49] | [0.01, 1] | [6.81, 16.31] | [442, 2304] |
| *Q. faginea* | 197.09 ± 1.383 | 7.15 ± 0.0649 | 0.71 ± 0.0028 | 11.3 ± 0.0135 | 782.23 ± 1.8832 |
|  | [75, 1598] | [0, 48.84] | [0.01, 1] | [6.7, 18.54] | [407, 1533] |
| *F. sylvatica* | 289.62 ± 1.1694 | 14.88 ± 0.0745 | 0.86 ± 0.0016 | 8.99 ± 0.0105 | 1191.7 ± 1.9639 |
|  | [75, 1649] | [0, 79.51] | [0.01, 1] | [4.16, 13.98] | [553, 2461] |
