# Supplementary material for: Patterns and Drivers of Tree Mortality in Iberian Forests: Climatic Effects Are Modified by Competition
Source: PLoS One. 2013 Feb 25;8(2):e56843. doi: 10.1371/journal.pone.0056843 (PMC3581527; doi:10.1371/journal.pone.0056843)
Supplement: Table S4 — Temperature and precipitation in cool vs. hot and dry vs. wet sites, respectively. Mean [minimum, maximum] values of mean annual temperature in cool and hot sites and annual precipitation in dry and wet sites are given for the 11 species included in the best model. (DOC) [file pone.0056843.s006.doc]

**Table S4. Temperature and precipitation in cool vs. warm and dry vs. wet sites, respectively.** Mean [minimum, maximum] values of mean annual temperature in cool and warm sites and annual precipitation in dry and wet sites are given for the 11 species included in the best model.

| Species | **Cool** | **Warm** | **Dry** | **Wet** |
| --- | --- | --- | --- | --- |
| *P. halepensis* | 12.41 | 15.59 | 387 | 753 |
|  | [9.95, 13.02] | [14.80, 19.21] | [170, 443] | [643, 1503] |
| *P. pinea* | 11.64 | 16.87 | 426 | 820 |
|  | [9.12, 12.12] | [15.68, 18.56] | [312, 462] | [732, 1682] |
| *P. pinaster* | 9.91 | 14.54 | 496 | 1310 |
|  | [7.69, 10.67] | [13.34, 18.46] | [322, 589] | [987, 3058] |
| *P. nigra* | 9.36 | 12.49 | 575 | 1052 |
|  | [6.24, 9.97] | [11.87, 15.62] | [360, 644] | [863, 2334] |
| *P. sylvestris* | 7.10 | 10.93 | 726 | 1249 |
|  | [3.43, 7.98] | [10.00, 14.72] | [428, 816] | [1079, 1977] |
| *P. uncinata* | 4.14 | 7.34 | 997 | 1470 |
|  | [1.81, 5.01] | [6.66, 14.20] | [521, 1095] | [1324, 1975] |
| *Q. ilex* | 10.36 | 16.26 | 515 | 1015 |
|  | [6.60, 11.25] | [15.32, 18.67] | [268, 585] | [852, 1988] |
| *Q. suber* | 13.59 | 17.21 | 664 | 1160 |
|  | [10.07, 14.45] | [16.35, 19.03] | [426, 729] | [1000, 1756] |
| *Q. pyrenaica* | 8.67 | 12.87 | 661 | 1257 |
|  | [6.81, 9.45] | [11.62, 16.31] | [442, 757] | [1108, 2304] |
| *Q. faginea* | 9.74 | 13.23 | 543 | 1061 |
|  | [6.70, 10.37] | [12.03, 18.54] | [407, 616] | [919, 1533] |
| *F. sylvatica* | 7.09 | 10.91 | 850 | 1573 |
|  | [4.16, 7.77] | [10.08, 13.98] | [553, 972] | [1401, 2461] |
