# Supplementary material for: Patterns and Drivers of Tree Mortality in Iberian Forests: Climatic Effects Are Modified by Competition
Source: PLoS One. 2013 Feb 25;8(2):e56843. doi: 10.1371/journal.pone.0056843 (PMC3581527; doi:10.1371/journal.pone.0056843)
Supplement: Table S5 — Bayesian means and 95% credible intervals for each parameter of the final model for each of the 11 common species. (DOC) [file pone.0056843.s007.doc]

**Table S5. Bayesian means and 95% credible intervals for each parameter of the final model for each of the 11 common species.**

|  |  | ***P. halepensis*** | ***P. pinea*** | ***P. pinaster*** | ***P. nigra*** | ***P. sylvestris*** | ***P. uncinata*** | ***Q. ilex*** | ***Q. suber*** | ***Q. pyrenaica*** | ***Q. faginea*** | ***F. sylvatica*** |
| --- | --- | --- | --- | --- | --- | --- | --- | --- | --- | --- | --- | --- |
| $\underline{\alpha}$ | **Mean** | -4.4039 | -4.5538 | -4.0574 | -5.2725 | -5.1069 | -5.5036 | -5.7897 | -5.1728 | -5.0140 | -5.8649 | -5.5282 |
|  | **CI** | (-4.4055, -4.4023) | (-4.5568, -4.5507) | (-4.0583, -4.0565) | (-5.2775, -5.2675) | (-5.1082, -5.1056) | (-5.5091, -5.498) | (-5.7927, -5.7867) | (-5.1756, -5.1699) | (-5.0171, -5.0109) | (-5.869, -5.8608) | (-5.531, -5.5253) |
|  | **Mean** | 0.8004 | 0.1317 | 0.1302 | 0.0336 | 0.0542 | -1.9795 | 0.0315 | 1.0605 | 0.1040 | 0.0709 | 0.4041 |
|  | **CI** | (0.7788, 0.8219) | (0.1295, 0.1338) | (0.1295, 0.1308) | (0.0335, 0.0337) | (0.0538, 0.0546) | (-2.0803, -1.8788) | (0.0313, 0.0316) | (1.0412, 1.0799) | (0.1031, 0.1049) | (0.0696, 0.0722) | (0.3881, 0.4200) |
|  | **Mean** | -0.0598 | -0.0287 | -0.0295 | -0.0095 | -0.0228 | -3.0256 | -0.0141 | -0.0526 | -0.0254 | -0.0235 | -0.0444 |
|  | **CI** | (-0.0601, -0.0594) | (-0.0288, -0.0285) | (-0.0295, -0.0294) | (-0.0096, -0.0095) | (-0.0228, -0.0227) | (-3.101, -2.9501) | (-0.0141, -0.014) | (-0.0529, -0.0524) | (-0.0255, -0.0253) | (-0.0237, -0.0233) | (-0.0448, -0.0439) |
| $\underline{\gamma_{1}}$ | **Mean** | 0.2847 | 0.3044 | 0.1708 | 0.1629 | 0.4030 | 0.1250 | 0.0278 | -0.0532 | 0.3163 | 0.0833 | 0.1294 |
|  | **CI** | (0.2835, 0.2859) | (0.3024, 0.3065) | (0.1703, 0.1714) | (0.1618, 0.164) | (0.4023, 0.4038) | (0.1212, 0.1289) | (0.0263, 0.0292) | (-0.0555, -0.0509) | (0.3149, 0.3176) | (0.0805, 0.0861) | (0.1276, 0.1313) |
| $\underline{\gamma_{2}}$ | **Mean** | 0.0850 | -0.0217 | 0.0144 | 0.0336 | -0.0174 | 0.0620 | 0.0298 | 0.1207 | -0.0155 | 0.0409 | 0.0340 |
|  | **CI** | (0.0840, 0.0859) | (-0.0227, -0.0206) | (0.0142, 0.0146) | (0.0332, 0.034) | (-0.0176, -0.0172) | (0.0614, 0.0626) | (0.029, 0.0306) | (0.1194, 0.122) | (-0.0161, -0.0149) | (0.0393, 0.0426) | (0.0334, 0.0345) |
| $\underline{\gamma_{3}}$ | **Mean** | -0.1162 | -0.0667 | -0.2647 | -0.0886 | 0.0166 | -0.0891 | -0.1539 | 0.0104 | 0.0312 | -0.2398 | 0.1647 |
|  | **CI** | (-0.1177, -0.1147) | (-0.0688, -0.0645) | (-0.2655, -0.2639) | (-0.0900, -0.0872) | (0.0155, 0.0176) | (-0.0924, -0.0857) | (-0.1554, -0.1524) | (0.0077, 0.0131) | (0.0293, 0.0331) | (-0.2428, -0.2367) | (0.1620, 0.1675) |
| $\underline{\gamma_{4}}$ | **Mean** | -0.0185 | -0.0518 | -0.0580 | -0.0898 | 0.0427 | 0.0131 | -0.0455 | 0.0186 | 0.0079 | -0.0152 | 0.0730 |
|  | **CI** | (-0.0192, -0.0178) | (-0.0528, -0.0508) | (-0.0584, -0.0576) | (-0.0905, -0.0891) | (0.0423, 0.0432) | (0.0117, 0.0146) | (-0.0461, -0.0449) | (0.0175, 0.0198) | (0.0071, 0.0087) | (-0.0162, -0.0141) | (0.0719, 0.0741) |
| $\underline{\gamma_{5}}$ | **Mean** | 0.0802 | -0.1282 | 0.1092 | 0.8754 | 0.1061 | -0.4947 | 0.1589 | -0.3551 | 0.0902 | 0.3141 | -0.0046 |
|  | **CI** | (0.0792, 0.0813) | (-0.1298, -0.1265) | (0.1087, 0.1098) | (0.8741, 0.8767) | (0.1055, 0.1067) | (-0.4996, -0.4899) | (0.1578, 0.1601) | (-0.3574, -0.3529) | (0.0892, 0.0912) | (0.3117, 0.3165) | (-0.0063, -0.0028) |
| $\underline{\gamma_{6}}$ | **Mean** | -0.0757 | 0.0131 | -0.0742 | -0.6029 | -0.0337 | -0.0899 | 0.1149 | 0.0629 | -0.0529 | 0.0330 | 0.0171 |
|  | **CI** | (-0.0767, -0.0746) | (0.0116, 0.0147) | (-0.0747, -0.0736) | (-0.6044, -0.6014) | (-0.0342, -0.0332) | (-0.0913, -0.0884) | (0.1138, 0.1161) | (0.061, 0.0649) | (-0.0542, -0.0516) | (0.0302, 0.0358) | (0.0157, 0.0185) |
| $\underline{\gamma_{7}}$ | **Mean** | 0.2197 | -0.0561 | 0.0524 | 0.0608 | 0.0294 | -0.2170 | 0.0641 | -0.1497 | -0.0307 | 0.0047 | 0.0604 |
|  | **CI** | (0.2189, 0.2206) | (-0.0576, -0.0547) | (0.0520, 0.0528) | (0.0600, 0.0616) | (0.0287, 0.03) | (-0.2213, -0.2126) | (0.0629, 0.0653) | (-0.1518, -0.1477) | (-0.0317, -0.0296) | (0.0024, 0.0069) | (0.0581, 0.0626) |
| $\underline{\gamma_{8}}$ | **Mean** | 0.0117 | -0.0298 | 0.0278 | -0.0152 | 0.0297 | 0.1503 | -0.0386 | -0.0597 | 0.0396 | -0.0535 | -0.0606 |
|  | **CI** | (0.0110, 0.0125) | (-0.0314, -0.0282) | (0.0277, 0.028) | (-0.0159, -0.0145) | (0.0292, 0.0301) | (0.1484, 0.1521) | (-0.0395, -0.0378) | (-0.0619, -0.0576) | (0.0388, 0.0404) | (-0.0559, -0.0512) | (-0.0621, -0.0592) |
| $\underline{\gamma_{9}}$ | **Mean** | 0.1370 | 0.0809 | -0.0136 | -0.1249 | -0.0064 | -0.0296 | 0.0322 | -0.0120 | 0.0112 | 0.0092 | 0.0681 |
|  | **CI** | (0.1362, 0.1377) | (0.0796, 0.0822) | (-0.014, -0.0132) | (-0.1259, -0.124) | (-0.0070, -0.0058) | (-0.031, -0.0281) | (0.0313, 0.0331) | (-0.0136, -0.0104) | (0.0101, 0.0123) | (0.0072, 0.0112) | (0.0668, 0.0694) |
| $\underline{\gamma_{10}}$ | **Mean** | -0.0118 | 0.0044 | 0.0055 | -0.1134 | -0.0205 | -0.0290 | 0.0047 | -0.0069 | -0.0404 | 0.0051 | 0.0204 |
|  | **CI** | (-0.0121, -0.0115) | (0.0038, 0.0049) | (0.0053, 0.0057) | (-0.1139, -0.113) | (-0.0208, -0.0203) | (-0.0295, -0.0284) | (0.0044, 0.0051) | (-0.0075, -0.0063) | (-0.0409, -0.0399) | (0.0043, 0.006) | (0.0197, 0.0211) |
| $\underline{\gamma_{11}}$ | **Mean** | 0.1581 | -0.0065 | 0.0087 | -0.0857 | 0.0129 | 0.0288 | 0.0729 | -0.2072 | 0.1054 | 0.1330 | 0.0591 |
|  | **CI** | (0.1567, 0.1594) | (-0.0086, -0.0045) | (0.0083, 0.0091) | (-0.0866, -0.0849) | (0.0126, 0.0133) | (0.0272, 0.0303) | (0.0714, 0.0744) | (-0.2095, -0.205) | (0.1044, 0.1064) | (0.1301, 0.1359) | (0.0581, 0.0601) |
| $\underline{\gamma_{12}}$ | **Mean** | 0.0699 | -0.0804 | -0.0096 | -0.0015 | -0.0182 | -0.0553 | 0.0440 | -0.1212 | -0.0666 | 0.0197 | -0.0139 |
|  | **CI** | (0.0690, 0.0709) | (-0.082, -0.0787) | (-0.0099, -0.0093) | (-0.0023, -0.0008) | (-0.0186, -0.0179) | (-0.0568, -0.0538) | (0.0425, 0.0454) | (-0.1236, -0.1189) | (-0.0675, -0.0657) | (0.0171, 0.0224) | (-0.0151, -0.0126) |
