# Supplementary material for: Patterns and Drivers of Tree Mortality in Iberian Forests: Climatic Effects Are Modified by Competition
Source: PLoS One. 2013 Feb 25;8(2):e56843. doi: 10.1371/journal.pone.0056843 (PMC3581527; doi:10.1371/journal.pone.0056843)
Supplement: Appendix S1 — Description of biotic and abiotic variables included in the analysis and variable selection. (DOCX) [file pone.0056843.s008.docx]

**Appendix S1.** **Description of abiotic and biotic variables included in the analysis and variable selection.**

Each tree was associated with six biotic variables related with stand structure, and 30 geographical variables (four topographic, 22 climatic and four edaphic). Each record measured in the SFI-2 was also characterized by six different biotic variables: *D* (diameter at breast height, mm), species dominance index (the basal area of a species divided by the total stand basal area in the stand), stand tree density (trees/ha), stand basal area (m^2^/ha), diameter coefficient of variation and stand basal area of larger trees (m^2^/ha). Although fire frequency had an important role in the Mediterranean [1,2], we did not include fire effects because we do not have reliable data at tree level to assess its effects. Moreover, plots which suffered catastrophic fires and were completely burned between the inventories were not included in the analysis because we only included permanent plots with trees that were alive in both consecutive inventories.

The four *topographic* variables were altitude (m), slope (degrees), aspect (degrees) and insolation (hours/year), interpolated from a STRM V1 digital elevation model with a 1 km^2^ resolution (Shuttle Radar Topographic Mission, <http://www2.ipl.nasa.gov/strm/>). The 22 *climatic* variables, calculated from Gonzalo [3] on a 1 km^2^ resolution, were annual precipitation (mm); seasonal precipitation (i.e., spring, summer, autumn and winter); mean annual temperature (ºC); seasonal temperature (ºC); mean annual temperature of the minimum and maximum (ºC); mean temperature of the warmest month (ºC); mean temperature of maxima in month with highest mean (ºC); mean temperature of the coldest month (ºC); mean temperature of the minimum in month with lowest mean (ºC); annual potential evapotranspiration [4]; annual surplus (in mm, calculated as the sum of positive differences between annual precipitation and potential evapotranspiration); annual deficit (in mm, calculated as the sum of negative differences between annual precipitation and potential evapotranspiration); thermal oscillation (ºC, calculated as the difference between mean maximum and mean minimum monthly temperature), drought length (calculated as the number of months in which potential evapotranspiration exceeded precipitation) and the vegetative active period according to Gaussen. Finally, the four *edaphic* variables*,* obtained from SFI-3, were rockiness (with 3 classes: 0-25%, 25-50%, >50%); organic matter content (highly humiferous, moderately humiferous, or non-humiferous soils); soil texture (sandy, loam and clay); and soil pH (acid (pH ≤ 6), neutral (pH = 7) or basic (pH ≥ 8)).

To select which indicator of competition performed better we compared single-predictor models which individually used basal area of larger trees, stand basal area, stand tree density and tree diameter coefficient of variation as predictors of mortality. We parameterised models with species-specific parameters using the non-linear functional form (Eqns. (1),(4)). The best predictor of competition (according to Bayesian Information Criteria, BIC) was basal area of larger trees and was retained for our modeling analysis (Table S1.1).

**Table S1.1.** Comparison of mortality models based on BIC parameterized at species level for different variables that could be used as proxy of competition. Predictor variables are B_L_ (basal area of larger trees), DCV (tree diameter coefficient of variation), BA (stand basal area) and SD (stand tree density). Number of parameter (NP), Bayesian Information Criterion (BIC) and ∆BIC are also shown.

| **Predictor variable** | **NP** | **BIC** | **∆BIC** |
| --- | --- | --- | --- |
| B_L_ | 327 | 322415 | **0** |
| DCV | 327 | 326818 | **4402** |
| BA | 327 | 326914 | **4429** |
| SD | 327 | 326844 | **4499** |

Our climatic and topographic datasets contained a large number of possible predictor variables which could influence mortality but which were likely to be correlated. A principal component analysis (PCA) was used to perform a variable reduction on the initial set of 26 different topographic and climatic variables highly correlated. The first two PCA axes explained 78.8% of the variance. The first axis of the PCA (explaining 53.8% of the variance) was strongly correlated with mean annual temperature and the second axis with annual precipitation (explaining 25.0% of the variance). Therefore, these two variables were selected for our modeling analysis as being representative of the variability in climatic conditions within the data (Fig. S2).

To select which edaphic variable performed better we tested one-dimensional models of the four edaphic variables (organic matter content, rockiness, soil texture and soil pH); parameterised with species-specific parameters and a non-linear functional form (Eqns. (1),(4)). The best predictor of edaphic conditions was organic matter content (see Table S1.2) and was retained for testing as a predictor of mortality with the other variables.

**Table S1.2.** Comparison of mortality models based on BIC parameterized at species level for different edaphic variables. Predictor variables are OMC (organic matter content), R (rockiness), TEX (soil texture) and pH (soil pH). Number of parameter (NP), Bayesian Information Criterion (BIC) and ∆BIC are also shown.

| **Predictor variable** | **NP** | **BIC** | **∆BIC** |
| --- | --- | --- | --- |
| **OMC** | 327 | 326692 | **0** |
| R | 327 | 327167 | **475** |
| TEX | 327 | 327215 | **523** |
| pH | 327 | 327399 | **707** |

Probability of mortality was parameterised for the 11 species present in the 46,678 plots for each potential predictor individually (6 predictors: three biotic variables, two climatic and one edaphic) to test the best functional form and parameter specification (global vs. species-specific parameters). We tested a total of 22 models parameterised with one predictor variable (11 at species-specific level and 11 considering all species together); including *D* in its exponential form and the rest of predictor variables in linear and non-linear form (Table S1). Comparing BIC values associated with each model allowed us to determine the significance of each predictor variable based on 10-units of difference (i.e. a ∆BIC>10 indicates significant support for the model with the lower BIC), and in what form it ought to be included in the model (global or species-specific parameters, linear or non-linear functional forms). The models with the smallest value of BIC had predictors in non-linear and species-specific forms, so we decided to retain those.

**References**

1. Verdú M, Pausas JG (2007) Fire drives phylogenetic clustering in Mediterranean Basin woody plant communities. J Ecol 95: 1316-1323.
2. Pausas JG (2004) Changes in fire and climate in the Eastern Iberian peninsula (Mediterranean basin). Clim Change 63: 337-350.
3. Gonzalo J (2008) Diagnosis fitoclimática de la España peninsular. Actualización y análisis geoestadístico aplicado. Madrid: Universidad Politécnica de Madrid. Escuela Técnica Superior de Ingenieros de Montes. 559 p.
4. Thornthwaite CW (1948) An approach toward a rational classification of climate. Geogr Rev 38: 55-94.
